# A comprehensive analysis of microRNA alteration in an ApoE(−/−) mice model of white adipose tissue injury induced by chronic intermittent hypoxia

**DOI:** 10.3389/fgene.2025.1474223

**Published:** 2025-03-26

**Authors:** Jinjie Zhang, Yaopeng Guo, Meilin Ji, Shu Lin, Dexin Liu, Qingshi Chen

**Affiliations:** ^1^ The Second Clinical Medical College, Fujian Medical University, Quanzhou, China; ^2^ The Second Affiliated Hospital of Fujian Medical University, Quanzhou, China; ^3^ Department of Endocrinology and Metabolism, The Second Affiliated Hospital of Fujian Medical University, Quanzhou, China; ^4^ Department of Interventional Radiology, The Second Affiliated Hospital of Fujian Medical University, Quanzhou, China

**Keywords:** microRNA, chronic intermittent hypoxia, white adipose tissue dysfunction, miRNA sequencing, bioinformatics analysis

## Abstract

**Background:**

MicroRNAs (miRNAs) represent a class of noncoding small RNAs and are implicated in many diseases. However, the role of miRNA in obstructive sleep apnea (OSA)-induced white adipose tissue (WAT) dysfunction remains to be fully elucidated. Using miRNA sequencing (miRNA-seq), we uncovered the miRNA expression profiles in chronic intermittent hypoxia (CIH)-induced WAT dysfunction mice.

**Methods:**

We established an apolipoprotein-deficient (ApoE−/−) CIH mouse model and identified differentially expressed miRNAs (DEmiRs) using miRNA-seq technology. With the help of Gene Ontology (GO) functional enrichment and the Kyoto Encyclopedia of Genes and Genomes (KEGG) pathway analyses, we determined the biological functions of these DEmiRs. In addition, RT-qPCR was performed for further evaluation of the sequencing data. Finally, we constructed a conserved negative correlation (CNC) network to expound the relationship between miRNA and target genes.

**Results:**

Overall, 13 miRNAs were found to be upregulated and 18 miRNAs downregulated in the CIH-induced mouse model of WAT dysfunction. KEGG pathway analysis results indicated that the lysosome pathway participated in CIH-induced WAT dysfunction. Then, eight miRNAs were shortlisted for RT-qPCR validation. Based on the data, we chose these DEmiRs to construct a miRNA–mRNA regulatory network.

**Conclusion:**

Overall, we identified 31 DEmiRs in the ApoE−/− CIH mouse model. Our findings may play a major role in explaining the pathophysiological mechanisms of WAT dysfunction induced by obstructive sleep apnea.

## 1 Introduction

Obstructive sleep apnea (OSA), a sleep disorder syndrome characterized by upper respiratory collapse during sleep, which induces chronic intermittent hypoxia (CIH) in the body. Previous studies have shown that the distribution proportion of OSA in middle-aged men and women is 34% and 17%, respectively (Yeghiazarians et al., 2021). OSA often leads to several complications, such as neuroinflammation (Liu et al., 2020), cardiac diseases (Bouzerda, 2018; Diamond and Ismail, 2021; Song et al., 2020), metabolic diseases (Liguori et al., 2019; Parati et al., 2016), and changes in gut microbiota (Durgan, 2017; Kuvat et al., 2020; Lv et al., 2023). Although there has been great progress in people’s understanding of OSA nowadays, there are also far more complications related to OSA than imagined, such as white adipose tissue (WAT) dysfunction (Gozal et al., 2017a; Varela-Guruceaga et al., 2020). Furthermore, it is difficult to separate obesity from OSA, as obesity and OSA often overlap and enhance each other, further worsening the condition (Kuvat et al., 2020). To date, the molecular mechanisms of OSA-associated WAT dysfunction remain unclear.

MicroRNAs (miRNAs) are a class of small noncoding RNAs that function in posttranscriptional regulation of gene expression. They are powerful regulators of various cellular activities including cell growth, differentiation, development, and apoptosis. Recently, as posttranscriptional regulators, miRNAs have been found to play a key role in fibrosis and cirrhosis (Gao et al., 2022a; Hochreuter et al., 2022a; Zhou et al., 2014). Furthermore, miRNAs also participate in cardiovascular disease (Kim et al., 2018; Li et al., 2019). In addition, miR-125b-5p in exosomes derived from adipose stem cells mitigates ferroptosis in pulmonary microvascular endothelial cells through the Keap1/Nrf2/GPX4 pathway in sepsis-related lung injury (Shen et al., 2023). However, the mechanism and role of miRNA in OSA-induced WAT dysfunction has not been well elucidated yet.

In the current study, we first used miRNA-seq technology to identify the miRNA expression profile of CIH-induced WAT dysfunction in a mouse model. Then, Gene Ontology (GO) and KEGG analyses were utilized to analyze the differential expression of mRNA targeting function, followed by RT-qPCR validation. Finally, we constructed a regulatory network for miRNA–mRNA based on the above results. This study contributes to a more detailed description of the WAT dysfunction mechanism associated to OSA.

## 2 Methods

### 2.1 Animals

Male ApoE-deficient mice were acquired from Guangdong Yaokang Biotechnology Co., Ltd. All experiments were approved by the Institutional Animal Care and Use Committee of the Second Affiliated Hospital of Fujian Medical University, China.

### 2.2 CIH samples

The ApoE-deficient mice were randomly divided into the CIH and the control group. We adopted the modeling approach from our earlier study (Chen et al., 2019). The mice were placed in cages equipped with gas control systems, which could alter nitrogen, oxygen, and air concentrations. Next, we simulated CIH in mice by controlling the inspiratory oxygen fraction to decrease oxygen from normal levels (21%) to approximately 6% within 60 s, followed by reoxygenation to normal levels within the next 60 s. The duration of CIH treatment was 8 weeks, during which oxygen level was measured by an O_2_ concentration monitor.

### 2.3 microRNA extraction and sequencing

All RNAs were extracted from injured mice WAT of the CIH models. The extracted RNA samples were subjected to agarose gel electrophoresis and NanoDrop quality inspection and quantification, after which RNA-seq library was constructed. Its quality was determined using an Agilent 2100 Bioanalyzer. The mixed sequencing libraries of different samples were denatured with 0.1M NaOH to generate single-stranded DNA, which was then sequenced on an Illumina sequencer following the manufacturer’s instructions.

### 2.4 Bioinformatics data analysis

We used GO enrichment analysis and the KEGG pathway to analyze the advanced functions of differentially expressed miRNA (DEmiR)-targeted genes. GO analysis organizes genes into hierarchical categories and reveals gene regulatory networks based on biological processes and molecular functions. KEGG analysis offers an analysis of gene signal transduction and disease pathways, thereby providing a foundation for gene function and pathway research. These analyses were based on information from the GO resource (http://www.geneontology. org) and the KEGG database (http://www.genome.jp/kegg/), using Cytoscape 3.10.1 software. *P* < 0.05 was considered statistically significant enrichment.

### 2.5 RT-qPCR

First, we reverse-transcribed all samples from all RNA to cDNA. We selected U6 small nucleolar RNA as the internal control. Subsequently, RT-qPCR was performed three times. The expression of tissue miRNAs was revealed after CIH treatment by RT-qPCR. Gene expression was analyzed using the 2^−ΔΔCT^ method. The sequences of PCR primers were presented in Table 1.

**TABLE 1:** Primers used for RT-qPCR analysis.

Gene	Sequence (5’->3′)	Length (bp)
U6	F:5′GCTTCGGCAGCACATATACTAAAAT3′ R:5′CGCTTCACGAATTTGCGTGTCAT3′	89
mmu-miR-21c	GSP:5′GGGGGTTAGCTTATCAGACTG3′ R:5′GTGCGTGTCGTGGAGTCG3′	65
mmu-miR-411-3p	GSP:5′GGGGTATGTAACACGGTCCA3′ R:5′GTGCGTGTCGTGGAGTCG3′	64
mmu-miR-211-5p	GSP:5′GGTTCCCTTTGTCATCCT3′ R:5′CAGTGCGTGTCGTGGAG3′	64
mmu-miR-18a-3p	GSP:5′GGGAACTGCCCTAAGTGCTC3′ R:5′GTGCGTGTCGTGGAGTCG3′	65
mmu-miR-1843a-3p	GSP:5′GGGCTCTGATCGTTCACCTC3′ R:5′GTGCGTGTCGTGGAGTCG3′	64
mmu-miR-181b-1-3p	GSP:5′GGGGGCTCACTGAACAATG3′ R:5′GTGCGTGTCGTGGAGTCG3′	64
mmu-miR-9-3p	GSP:5′GGGGGGATAAAGCTAGATAACC3′ R:5′ GTGCGTGTCGTGGAGTCG3′	66
mmu-miR-450b-3p	GSP:5′GGGATTGGGAACATTTTGC3′ R:5′GTGCGTGTCGTGGAGTCG3′	63

### 2.6 Prediction of target genes of identified CIH-related miRNAs

In order to detect the potential functions of these DEmiRs, miRNA–mRNA networks were constructed using mRNAs predicted by at least two databases and a visualized network between miRNA and mRNA established using Cytoscape software.

### 2.7 Statistical analysis

Data were expressed as mean ± standard deviation (SD) from independent experiments and statistical analysis performed using SPSS (version 19.0) and Prism 7.0 (GraphPad). We compared the two groups using unpaired Student's t-test. Differences were considered significant at *p* < 0.05.

## 3 Results

### 3.1 Differential expression of miRNAs

**FIGURE 1:**
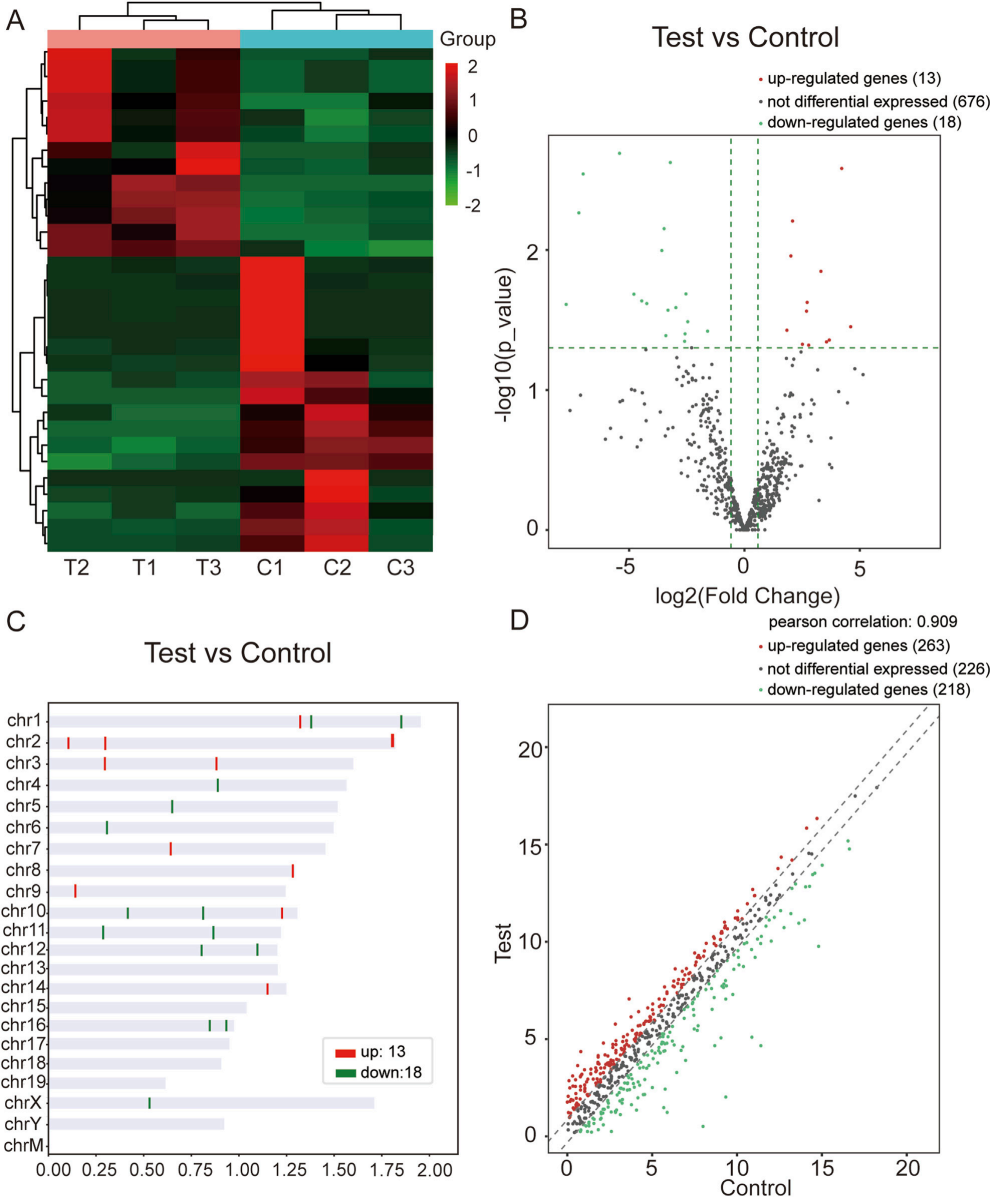
miRNA-seq data corresponding to the DEmiRs. **(A)** Hierarchical clustering analysis of DEmiRs in WAT from the CIH and control groups. The color scheme indicates upregulated miRNAs in red and downregulated miRNAs in green, allowing visualization of miRNA expression patterns across samples. **(B)** Volcano plot of DEmiRs. This plot highlights the most significant changes in miRNA expression between the CIH and control groups. Red dots represent upregulated miRNAs, while green dots represent downregulated ones. The X-axis represents the fold change, and the Y-axis represents the statistical significance of the changes (log10 of *P*-value). **(C)** Chromosomal distribution of DEmiRs. The figure shows the chromosomal locations of the upregulated (red) and downregulated (green) miRNAs. This distribution provides insights into the genomic context of these miRNAs within the mouse genome. **(D)** Scatter plot of gene expression variation between the CIH and control groups. The scatter plot illustrates the expression differences between the CIH and control groups. Red dots represent miRNAs that were upregulated in CIH, and green dots indicate those that were downregulated. The plot visually summarizes the overall gene expression changes.

Using miRNA-seq, we confirmed the DEmiRs in the mouse model of WAT dysfunction induced by CIH. We found 31 differentially expressed miRNAs, of which 13 were upregulated while 18 were downregulated. Hierarchical clustering analysis and volcano plot analysis of all differentially expressed miRNAs are displayed in Figure 1A, B, respectively. Chromosomal distribution of these DEmiRs is shown in Figure 1C. Scatter plots were utilized to illustrate gene expression variation between the CIH and control groups (Figure 1D).

### 3.2 GO and KEGG analysis

**FIGURE 2:**
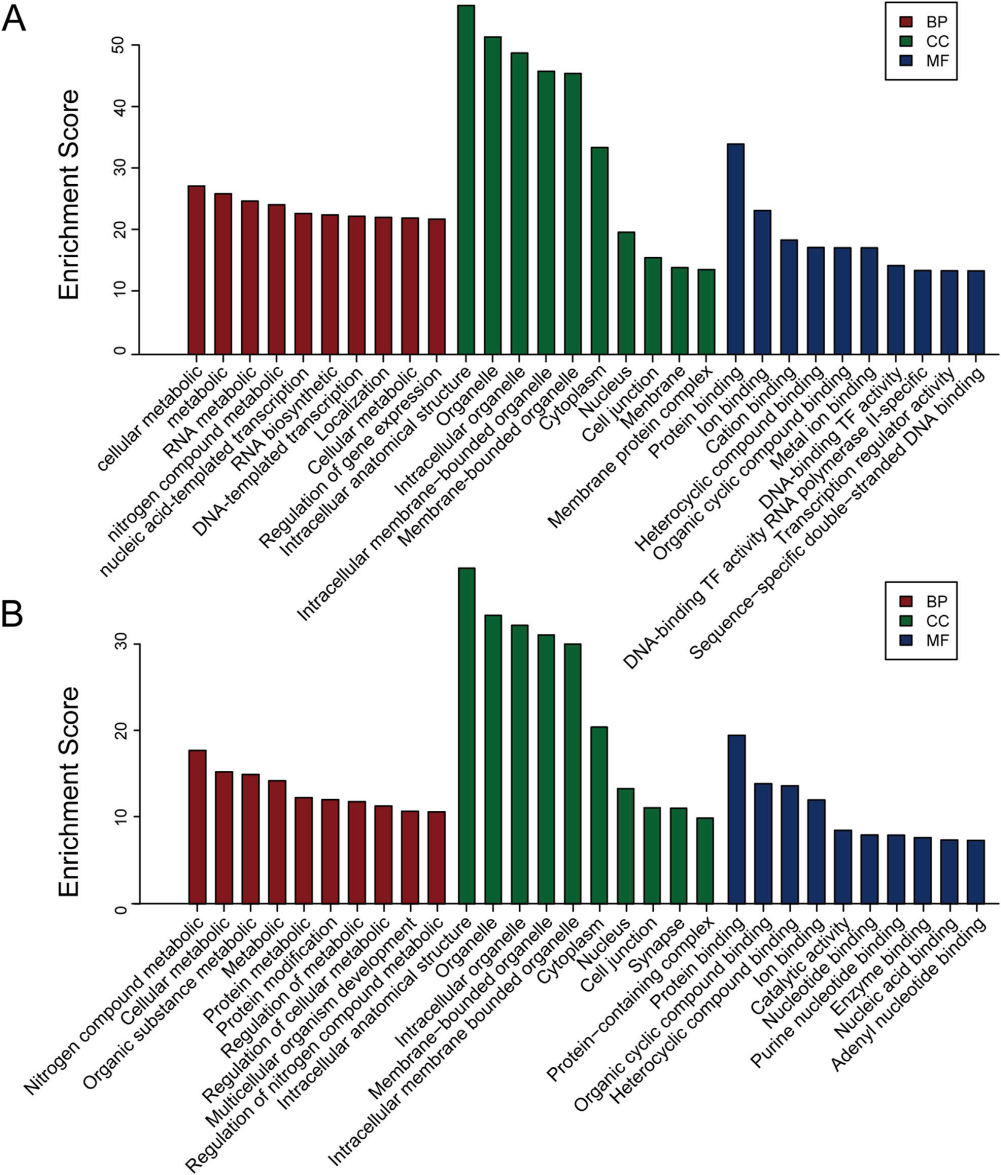
GO analysis of the target genes of DEmiRs. **(A)** GO analysis for the target genes of upregulated DEmiRs. This analysis categorizes the biological processes, cellular components, and molecular functions associated with the upregulated miRNAs. The most enriched GO terms suggest that these miRNAs may be involved in various metabolic and inflammatory processes in CIH-induced WAT dysfunction. **(B)** GO analysis of the target genes of downregulated DEmiRs. Similar to **A**, GO analysis of the downregulated miRNAs identifies biological pathways and functions potentially impacted by the reduction in miRNA expression. These findings are indicative of miRNA-mediated regulatory networks affecting cellular processes critical to WAT function.

**FIGURE 3:**
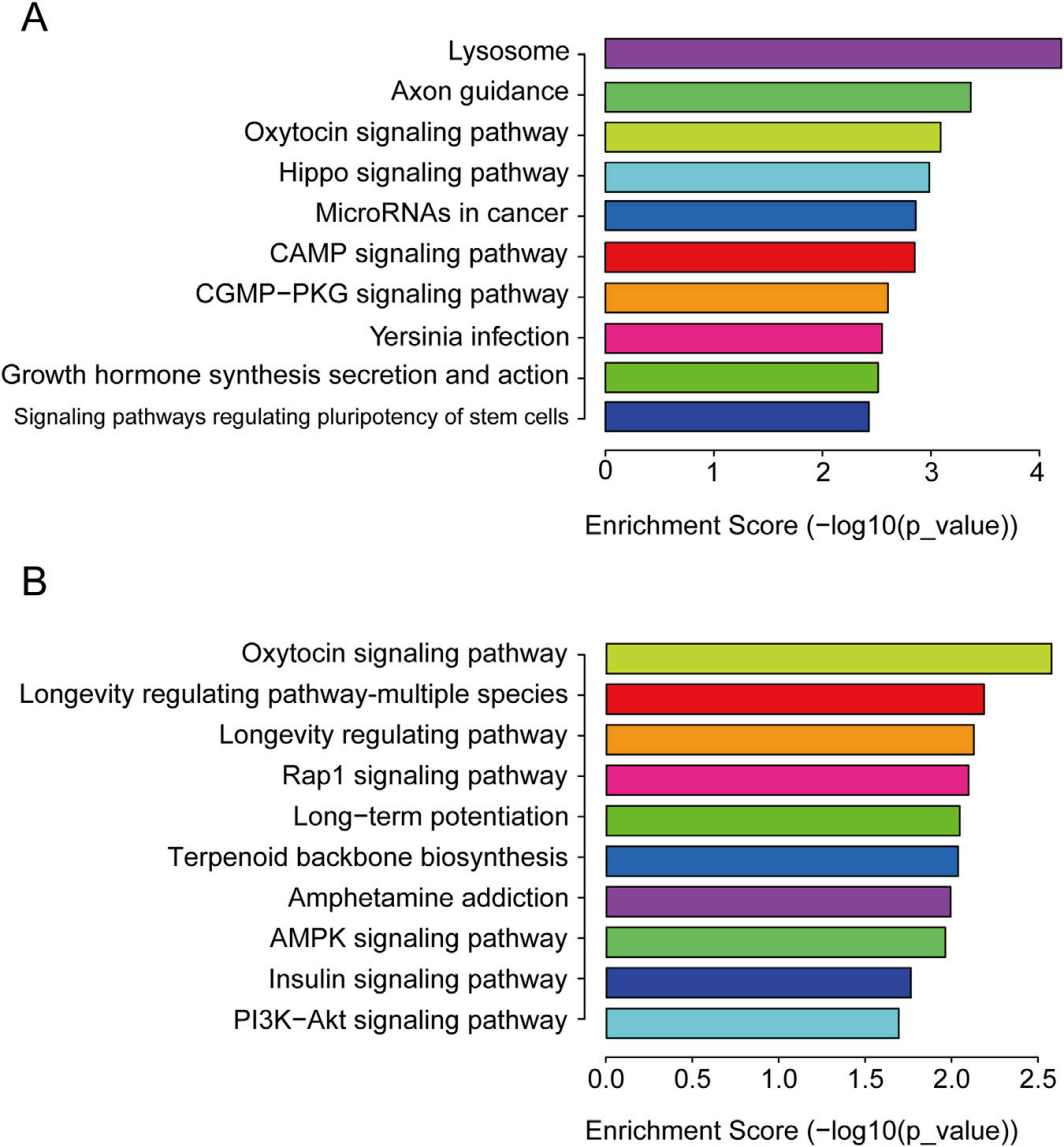
KEGG pathway. **(A)** KEGG pathway analysis of the target genes of upregulated DEmiRs. The top 10 pathways enriched for upregulated miRNAs are shown, highlighting key signaling pathways, such as the lysosome and insulin signaling pathways, that are likely disrupted in CIH-induced WAT dysfunction. These pathways are involved in cellular degradation, metabolism, and tissue remodeling. **(B)** KEGG pathway analysis of the target genes of downregulated DEmiRs. This panel shows the top 10 pathways enriched for downregulated miRNAs. The analysis reveals pathways involved in cellular stress response and lipid metabolism, which are crucial for understanding the metabolic alterations associated with WAT dysfunction under CIH conditions.

To better elucidate the potential function of DEmiRs in the pathogenesis of CIH-induced WAT dysfunction, we utilized GO analysis to make a detailed explanatory note of targeted genes. The results confirmed that these DEmiRs mainly participate in the following domains: biological process, cellular process, and molecular function (Figures 2A,B). All these findings demonstrated that these DEmiRs play specific roles in CIH-induced WAT dysfunction. In addition, we identified 71 KEGG pathways for all DEmiRs. The top 10 pathways of up- and downregulated DEmiRs are listed in Figures 3A, B, including the lysosome pathway, insulin signaling pathway, the CGMP–PKG signaling pathway, and the CAMP signaling pathway.

### 3.3 Validation of DEmiRs using RT-qPCR

**FIGURE 4:**
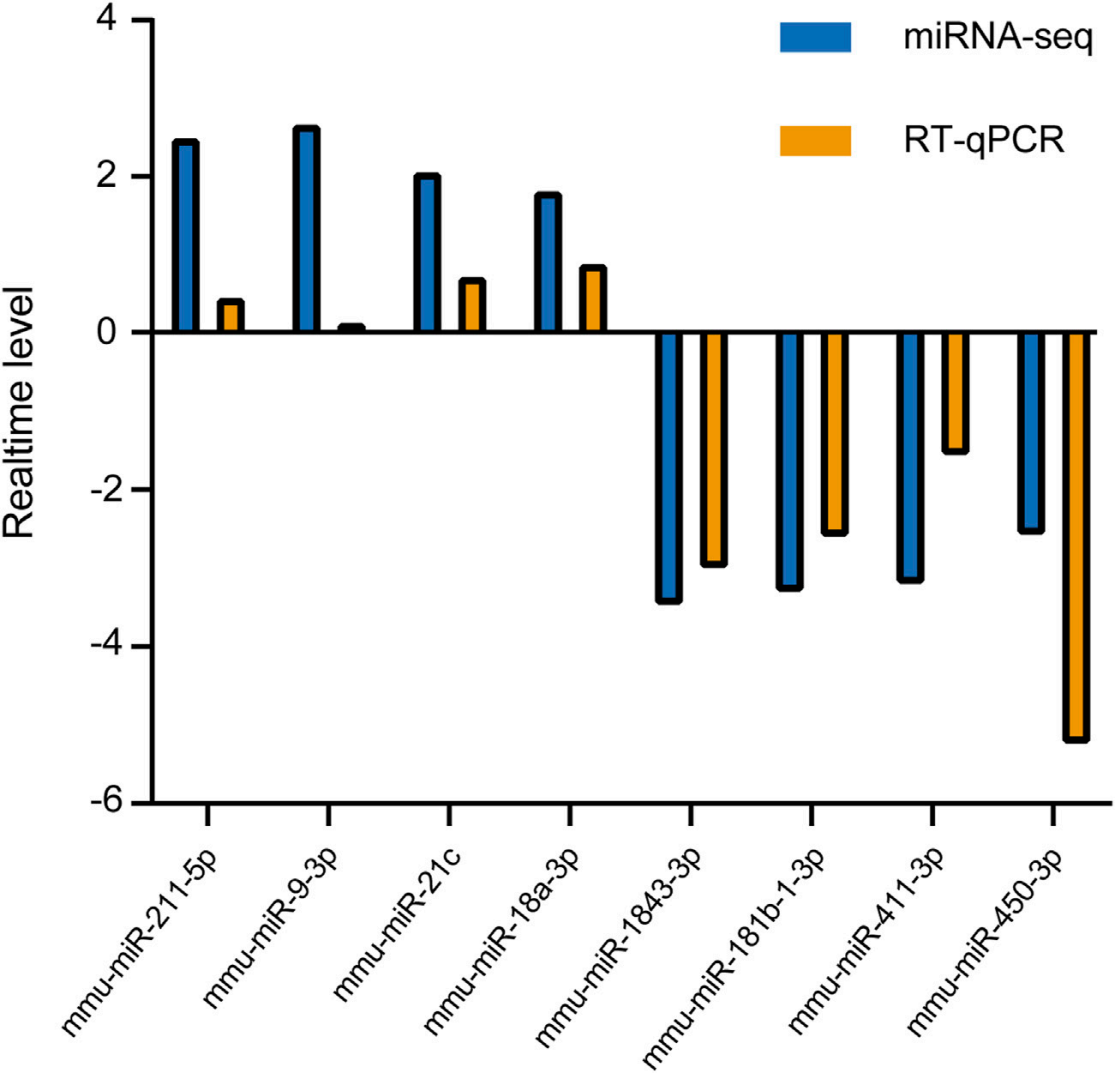
RT-qPCR validation of candidate DEmiRs. Four miRNAs (mmu-miR-211-5p, mmu-miR-9-3p, mmu-miR-21c, and mmu-miR-18a-3p) were upregulated, and four miRNAs (mmu-miR-1843a-3p, mmu-miR-181b-1-3p, mmu-miR-411-3p, and mmu-miR-450-3p) were downregulated in CIH-treated mice. The RT-qPCR data confirm the trends observed in miRNA-seq, supporting the reliability of the sequencing results. Each miRNA’s relative expression level was normalized to U6 and presented as fold change (mean ± SD).

Several DEmiRs were selected to determine the expression levels using RT-qPCR. The results showed that four RNAs (mmu-miR-211-5p, mmu-miR-9-3p, mmu-miR-21c, and mmu-miR-18a-3p) were upregulated and four RNAs (mmu-miR-1843a-3p, mmu-miR-181b-1-3p, mmu-miR-411-3p, and mmu-miR-450-3p) were downregulated, which was consistent with our sequencing results (Figure 4). This indicated reliability of the sequencing data results. However, the RT-qPCR results for mmu-miR-9-3p showed only a minimal increase, which is inconsistent with the miRNA-seq findings. This discrepancy may be attributed to the differences in sensitivity between the two techniques, and potential biological variability in the samples.

### 3.4 Construction of the miRNA–mRNA network

**FIGURE 5:**
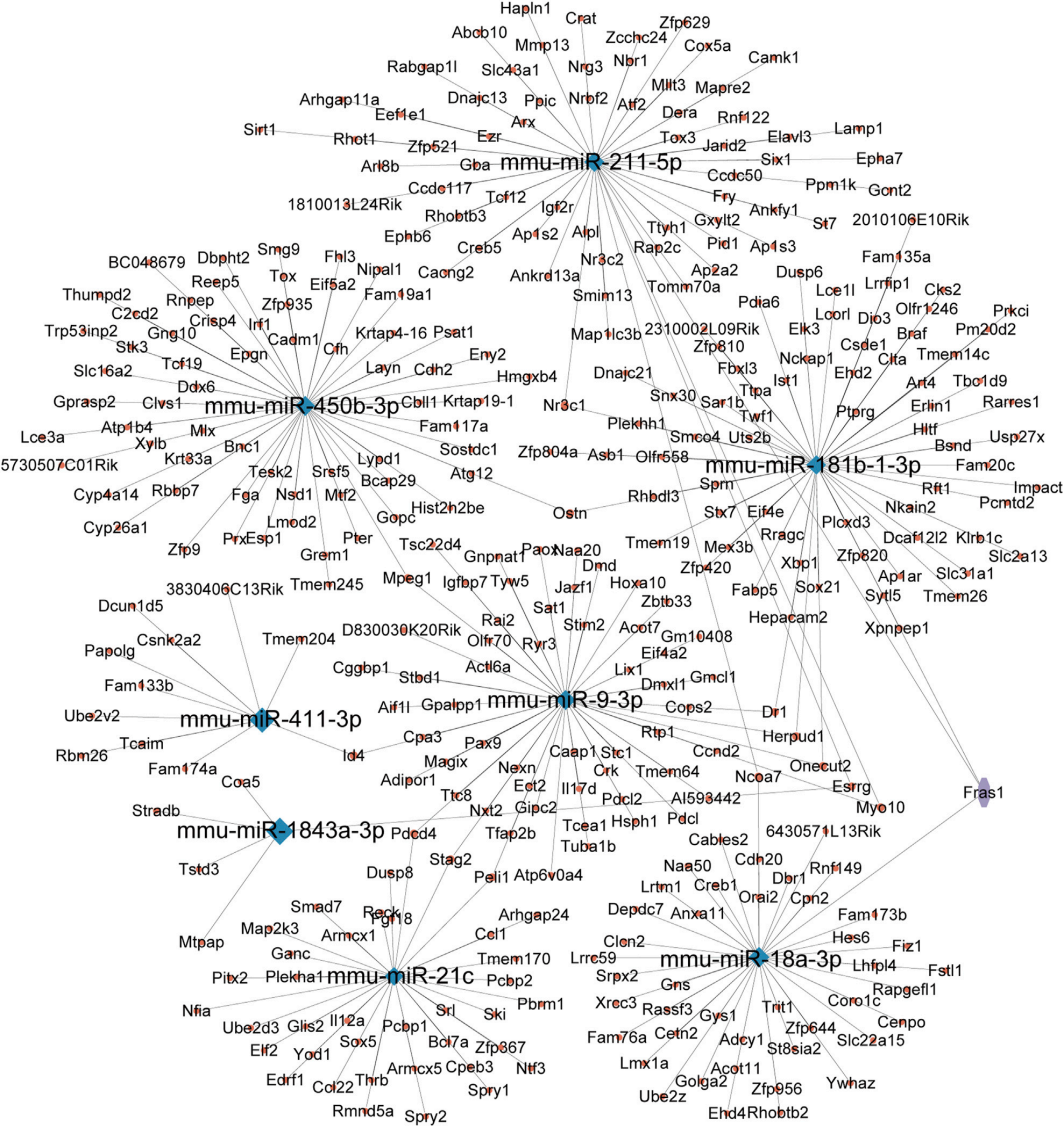
miRNA–mRNA interaction network. This network was constructed based on the predicted target genes of the five selected DEmiRs. Blue nodes represent the miRNAs, while red nodes represent the corresponding mRNAs. The edges between miRNAs and mRNAs indicate potential regulatory relationships, suggesting how these miRNAs might influence gene expression in the context of CIH-induced WAT dysfunction.

miRNAs for the miRNA–mRNA network construction were selected based on their statistical significance, fold-change values, and biological relevance, as determined through GO and KEGG pathway analyses. We chose eight miRNAs, mmu-miR-211-5p, mmu-miR-184b-1-3p, mmu-miR-450b-3p, mmu-miR-411-3p, mmu-miR-9-3p, mmu-miR-1843a-3p, mmu-miR-21c, and mmu-miR-18a-3p, and integrated them with the corresponding target genes into the miRNA–mRNA network created using the Cytoscape platform (Figure 5). Our research findings provided a new research strategy for exploring the potential mechanisms of these DEmiRs by revealing their targeted mRNA.

## 4 Discussion

To our knowledge, our current study reported for the first time a comprehensive analysis of the differential expression profile of miRNAs in a mouse model of WAT injury induced by CIH. We also systematically explained the potential functions and pathway enrichments through bioinformatics analysis. The results of the study could promote our understanding of how miRNA expression alteration acts in the unrevealed mechanism of OSA-related WAT dysfunction.

OSA has been considered one of the causes of several metabolic disorders. CIH, a key characteristic of OSA, plays an important role in the pathological process of the disease. Previous studies have proven that long-term CIH leads to insulin resistance in lean mice by durably altering the insulin signaling pathway in WAT (Murphy et al., 2017). Importantly, insulin resistance is the independent risk factor of OSA (Bros et al., 2018; Gao et al., 2022b). The ApoE−/− model is well-established in obesity-related pathophysiology, making it an ideal system for studying metabolic dysfunction in the context of CIH-induced WAT injury. Furthermore, the model has been shown to exhibit significant metabolic alterations, making it highly relevant to OSA-related research. Additionally, CIH can also lead to vascular rarefaction and inflammation in the body. Vascular rarefaction obstructs the nutrient acquisition of white adipose tissue, while inflammation leads to changes in the size, morphology, and dysfunction of white adipose tissue (Kawai et al., 2021). Recent studies suggest that continuous positive airway pressure (CPAP) may be beneficial for adipose tissue hormone levels among individuals with OSA (Zirlik et al., 2011). All these factors might result in WAT dysfunction. However, the exact mechanism between OSA and WAT dysfunction remains to be explored.

miRNAs represent a series of noncoding small RNAs and play key roles in the posttranscriptional regulation of gene expression. This suggests that miRNA may be a new perspective for understanding diseases, and many studies have indeed confirmed this, such as miRNAs acting as therapeutic targets for cardiovascular diseases (Zhou et al., 2018). Zhou et al.’s (2018) research showed that it is possible to prevent post-infraction remodeling and improve cardiac function by administering miR22 anti-miRNAs in elderly mice (Zhou et al., 2018). In addition, Kim et al. found that microRNA-21-mediated SATB1/S100A9/NF-κB axis promotes chronic obstructive pulmonary disease pathogenesis (Kim et al., 2021). However, few studies have combined miRNA and CIH-induced WAT dysfunction. Therefore, we took advantage of the ApoE-deficient mouse model to reveal role miRNAs play in the pathology of WAT dysfunction caused by CIH.

In the current study, 31 miRNAs were found to be differentially expressed during the process of CIH-induced WAT injury, of which 13 were upregulated and 18 were downregulated. The specific DEmiRs were evaluated by RT-qPCR, and the results were found to be consistent with miRNA-seq data. In addition, miRNAs may participate in diverse biological and pathological processes. For instance, miR-210 was significantly upregulated during hypoxia and played a protective role by inhibiting apoptosis and regulating cell proliferation, differentiation, migration, and angiogenesis in hypoxic cells (Guan et al., 2019). Additionally, research revealed that miR-133a participated in the early pathology of MI, and in subsequent cardiac remodeling (Xiao et al., 2019). Furthermore, the enlargement of miR-411-3p could inhibit cell proliferation and migration in lung fibroblasts with TGF-β1 treatment and attenuate lung fibrosis in silicotic mice (Gao et al., 2020). However, the roles of these DEmiRs in the development of CIH-induced WAT injury remain poorly understood.

We subjected these DEmiRs to GO and KEGG enrichment analyses in order to further determine their biological function in CIH-induced WAT dysfunction. The results showed that approximately 71 pathways might be regulated by these DEmiRs. For the upregulated DEmiRs, the lysosome pathway was the most enriched one. The autophagy–lysosomal degradation pathway plays a fundamental role in cellular, tissue, and organismal homeostasis. During autophagy, excess mitochondria in adipose tissue are eliminated, leading to the transformation of brown adipose tissue into white adipose tissue (Li and Ding, 2017). Excessive white adipose tissue could cause a series of tissue function disorders. Previous research have demonstrated that it is possible to ameliorate liver steatosis and fibrosis and to decrease serum FFA levels via adipose tissue–liver crosstalk under autophagy suppression conditions (Sakane et al., 2021). We subjected the GO and KEGG enrichment analyses of DEmiRs. Combining the results and previous studies, we concluded that the enriched lysosome pathway might participate in many disease procedures. Although lysosome pathways play important roles during the pathological process of CIH-induced WAT dysfunction, the exact mechanism remains to be fully understood. Simultaneously, our investigation into the realm of OSA disease has also yielded notable advancements, such as the CGMP-PKG signaling pathway, which may become a vital research direction in the future.

Thus, we predicted the assumed target of these DEmiRs using miRWalk and miRDB databases. The intricate mRNA networks demonstrated that the regulation of mRNA by miRNAs was not a one-to-one process. Clarifying the functions of these target genes could enrich our understanding of the complicated molecular mechanisms of WAT dysfunction induced by CIH. In this study, we found miR-211-5p could target Sirtuin 1 (SIRT1), a histone/protein deacetylase implicated in aging, metabolism, and stress resistance. SIRT1 regulates endothelial nitric oxide (NO) synthase, restores NO availability, and is involved in different aspects of cardiovascular disease. Previous research has shown that the blood level of SIRT1 has a strong association with OSA (Chen et al., 2015). Successful treatment for OSA with nasal CPAP can restore blood levels of the SIRT1 protein and its activity and serum levels of NOx. Therefore, up- or downregulation of the gene coding SIRT1 may be involved in the process of CIH-induced WAT dysfunction. It is therefore imperative to explore in more detail the function and molecular mechanism of these differentially expressed miRNAs in the future.

However, our study still has some limitations. First, we did not conduct cell-specific identification of these miRNAs. Their role in the disease has not yet been elucidated, which will be the focus of our next work. Second, we used only male ApoE-deficient mice in our study, which limits generalizability across different biological sexes. Including female mice would make the results more robust and applicable to a broader population. Third, the small sample size of the experiment makes it challenging to achieve universality of the current results. Fourth, the study lacks appropriate controls to evaluate the impact of ApoE deficiency independently of CIH-induced effects. Comparisons with wild-type mice under similar hypoxic conditions could help delineate the specific contributions of ApoE deficiency. Fifth, the criteria for selecting CIH-induced injury in WAT are based solely on miRNA expression profiles without consideration of gold standard metabolic or histological assessments. This may undermine our research, causing it to lack convincing persuasiveness.

In summary, our study for the first time used miRNA-seq technology to analyze DEmiRs in a CIH-induced WAT dysfunction mouse model. These findings have helped better understand OSA-related WAT dysfunction molecular etiology. In the future, further studies on the mechanism of these miRNAs will help provide new treatment strategies for this disease.

## Data Availability

The data presented in the study are deposited in the GEO repository, accession number GSE292464.

## Supplementary material

The Supplementary Material for this article can be found at: https://www.frontiersin.org/articles/10.3389/fgene.2025.1474223/full#supplementary-material
