# Supplementary material for: A comprehensive analysis of microRNA alteration in an ApoE(−/−) mice model of white adipose tissue injury induced by chronic intermittent hypoxia
Source: Front Genet. 2025 Mar 26;16:1474223. doi: 10.3389/fgene.2025.1474223 (PMC11979184; doi:10.3389/fgene.2025.1474223)
Supplement: Supplementary file 1 [file Table1.docx]

| **Gene** | **Sequence (5’->3’)** | **Length(bp)** |
| --- | --- | --- |
| U6 | F:5’GCTTCGGCAGCACATATACTAAAAT3’  R:5’CGCTTCACGAATTTGCGTGTCAT3’ | 89 |
| mmu-miR-21c | GSP:5’GGGGGTTAGCTTATCAGACTG3’  R:5’GTGCGTGTCGTGGAGTCG3’ | 65 |
| mmu-miR-411-3p | GSP:5’GGGGTATGTAACACGGTCCA3’  R:5'GTGCGTGTCGTGGAGTCG3' | 64 |
| mmu-miR-211-5p | GSP:5'GGTTCCCTTTGTCATCCT3’  R:5'CAGTGCGTGTCGTGGAG3' | 64 |
| mmu-miR-18a-3p | GSP:5’GGGAACTGCCCTAAGTGCTC3’  R:5’GTGCGTGTCGTGGAGTCG3’ | 65 |
| mmu-miR-1843a-3p | GSP:5’GGGCTCTGATCGTTCACCTC3’  R:5'GTGCGTGTCGTGGAGTCG3’ | 64 |
| mmu-miR-181b-1-3p | GSP:5’GGGGGCTCACTGAACAATG3’  R:5'GTGCGTGTCGTGGAGTCG3’ | 64 |
| mmu-miR-9-3p | GSP:5’GGGGGGATAAAGCTAGATAACC3'  R:5’ GTGCGTGTCGTGGAGTCG3’ | 66 |
| mmu-miR-450b-3p | GSP:5'GGGATTGGGAACATTTTGC3'  R:5'GTGCGTGTCGTGGAGTCG3' | 63 |

**Table1 Primers used for RT-qPCR.**
